# Tris(ethyl­enediamine-κ^2^ *N*,*N*′)nickel(II) bis­(dimethyl phosphate)

**DOI:** 10.1107/S1600536812029984

**Published:** 2012-07-07

**Authors:** Masoud Rafizadeh, Hamid Reza Saadati Moshtaghin, Vahid Amani

**Affiliations:** a Department of Chemistry, Share-Ray Branch, Islamic Azad University, Tehran, Iran

## Abstract

In the title compound, [Ni(C_2_H_8_N_2_)_3_][O_2_P(OCH_3_)_2_]_2_, the Ni^II^ atom is six-coordinated in a distorted octa­hedral geometry by six N atoms from three ethyl­enediamine ligands. The P atoms of the anions adopt a distorted tetra­hedral geometry. In the crystal, inter­molecular N—H⋯O and C—H⋯O hydrogen bonds link the cations and anions into a three-dimensional network.

## Related literature

For related structures, see: Amani *et al.* (2006 ▶); Jun & Zhang (2010 ▶); Rafizadeh & Amani (2006*a* ▶,*b* ▶, 2007 ▶); Rafizadeh, Amani & Aghayan (2006 ▶); Rafizadeh, Amani & Broushaky (2006 ▶); Rafizadeh, Hoseinzadeh & Amani (2006 ▶); Rafizadeh *et al.* (2005 ▶, 2007 ▶, 2009 ▶).
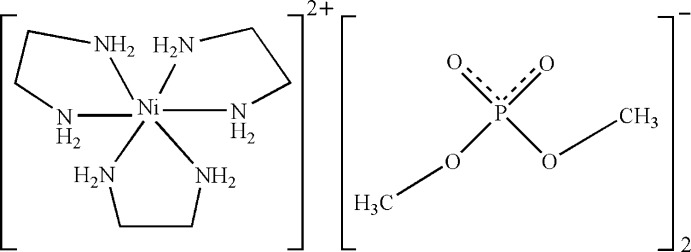


## Experimental

### 

#### Crystal data

[Ni(C_2_H_8_N_2_)_3_](C_2_H_6_O_4_P)_2_
*M* *_r_* = 489.08
Monoclinic, 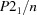

*a* = 9.2553 (5) Å
*b* = 12.4913 (5) Å
*c* = 18.190 (1) Å
β = 90.156 (4)°
*V* = 2102.95 (18) Å^3^
*Z* = 4
Mo *K*α radiation
μ = 1.12 mm^−1^
*T* = 120 K
0.49 × 0.40 × 0.38 mm

#### Data collection

Stoe IPDS-2T diffractometer
Absorption correction: numerical (*X-SHAPE* and *X-RED*; Stoe & Cie, 2002 ▶) *T*_min_ = 0.590, *T*_max_ = 0.650
9338 measured reflections
5205 independent reflections
4555 reflections with *I* > 2σ(*I*)
*R*_int_ = 0.031

#### Refinement

*R*[*F* ^2^ > 2σ(*F* ^2^)] = 0.034
*wR*(*F* ^2^) = 0.085
*S* = 1.08
5205 reflections
284 parameters
H atoms treated by a mixture of independent and constrained refinement
Δρ_max_ = 0.51 e Å^−3^
Δρ_min_ = −0.40 e Å^−3^

### 

Data collection: *X-AREA* (Stoe & Cie, 2002 ▶); cell refinement: *X-AREA*; data reduction: *X-RED* (Stoe & Cie, 2002 ▶); program(s) used to solve structure: *SHELXS97* (Sheldrick, 2008 ▶); program(s) used to refine structure: *SHELXL97* (Sheldrick, 2008 ▶); molecular graphics: *ORTEP-3* (Farrugia, 1997 ▶); software used to prepare material for publication: *WinGX* (Farrugia, 1999 ▶).

## Supplementary Material

Crystal structure: contains datablock(s) I, global. DOI: 10.1107/S1600536812029984/hy2566sup1.cif

Structure factors: contains datablock(s) I. DOI: 10.1107/S1600536812029984/hy2566Isup2.hkl

Additional supplementary materials:  crystallographic information; 3D view; checkCIF report

## Figures and Tables

**Table 1:** Hydrogen-bond geometry (Å, °)

*D*—H⋯*A*	*D*—H	H⋯*A*	*D*⋯*A*	*D*—H⋯*A*
N1—H1*C*⋯O2	0.90 (3)	2.17 (3)	3.057 (2)	171 (2)
N1—H1*D*⋯O5^i^	0.93 (4)	2.36 (4)	3.263 (2)	165 (3)
N2—H2*C*⋯O6	0.87 (3)	2.35 (3)	3.111 (2)	146 (2)
N2—H2*D*⋯O6^ii^	0.84 (3)	2.19 (3)	3.015 (2)	169 (2)
N3—H3*C*⋯O2^iii^	0.92 (3)	2.11 (3)	2.971 (2)	157 (2)
N3—H3*D*⋯O6	0.90 (3)	2.13 (3)	3.002 (2)	163 (2)
N4—H4*C*⋯O1	0.92 (3)	2.00 (3)	2.910 (2)	176 (3)
N4—H4*D*⋯O8^ii^	0.88 (3)	2.40 (3)	3.205 (2)	152 (2)
N5—H5*C*⋯O5^i^	0.87 (3)	2.11 (3)	2.911 (2)	154 (2)
N6—H6*C*⋯O4	0.90	2.35	3.243 (2)	175
N6—H6*D*⋯O2^iii^	0.90	2.20	3.064 (2)	160
C9—H9*C*⋯O1^iv^	0.96	2.41	3.305 (3)	155

Symmetry codes: (i) 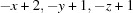; (ii) 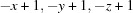; (iii) 
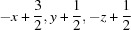
; (iv) 
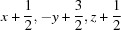
.

**Fig. 1.**
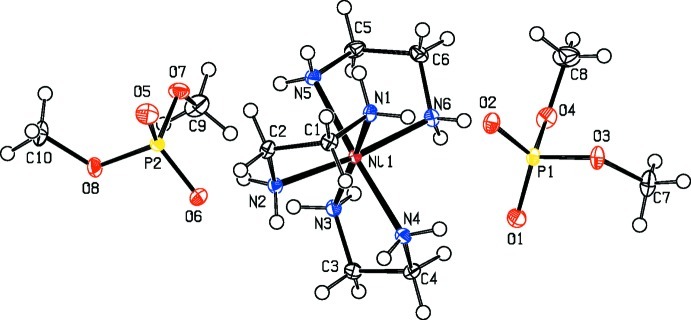
The molecular structure of the title compound. Displacement ellipsoids are drawn at the 30% probability level.

**Fig. 2.**
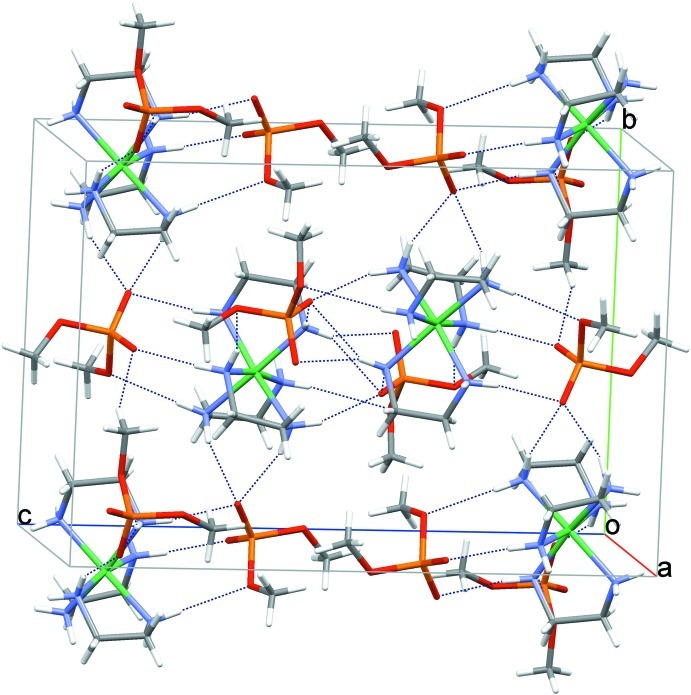
Crystal packing diagram for the title compound. Hydrogen bonds are shown as dashed lines.

## supplementary crystallographic information

### Comment

In recent years, we reported the synthesis and crystal structure of [Ni(H_2_O)_6_](DMP)_2_ (Rafizadeh & Amani, 2006*a*) (DMP = dimethylphosphate anion, [O_2_P(OCH_3_)_2_]^-^). In this compound, DMP is not bonded to the metal but acts as a counterion. Also, we reported the syntheses and crystal structures of [Cu_2_(µ-DMP)_4_(µ-DMSO)]_n_ (Rafizadeh *et al.*, 2005), [UO_2_(µ-DMP)_2_(DMSO)]_n_ (Rafizadeh, Hoseinzadeh & Amani, 2006), [La(µ-DMP)_2_(µ_3_-NO_3_)(DMSO)]_n_ (Rafizadeh, Amani & Broushaky, 2006), [UO_2_(µ-DEP)_2_(DMSO)]_n_ (Rafizadeh & Amani, 2006*b*), {[Mn_2_(µ-DMP)_3_(µ-DMSO)_2_(DMSO)(H_2_O)]NO_3_.H_2_O}_n_ (Rafizadeh, Amani, & Aghayan, 2006), [Ce_2_(µ-DEP)_6_(TEP)]_n_ (Amani *et al.*, 2006), [Mn_2_(µ_3_-DMP)_2_(µ-DMP)_2_]_n_ (Rafizadeh *et al.*, 2007), [Nd(µ-DEP)_3_]_n_ and [Pr(µ-DMP)_2_(µ-NO_3_)(DMSO)]_n_ (Rafizadeh *et al.*, 2009) (DMSO = dimethylsulfoxide, DEP = diethylphosphate and TEP = triethylphosphate). DMP and DEP act as O-donor ligands, thus forming coordination polymers in solid state. We have also reported the synthesis and crystal structure of [Fe_16_(µ_3_-O)_8_(µ_3_-OH)_4_(µ-OH)_4_(µ-DMP)_12_ (µ-OAc)_12_(DMSO)_4_].2DMSO.1.5H_2_O (Rafizadeh & Amani, 2007), which consists of sixteen iron ions connected by twelve bridging dimethylphosphates, twelve bridging acetates, eight µ_3_-oxo, four µ_3_-OH, four µ-OH groups and four DMSO ligands. We now report the synthesis and structure of the title compound, which was synthesized by the reaction of [Ni(H_2_O)_6_](DMP)_2_ and ethylenediamine (en).

In the title compound (Fig. 1), the Ni^II^ atom is six-coordinated in a distorted octahedral geometry by six N atoms from three en ligands. The Ni—N bond lengths and angles are within normal range as observed in [Ne(en)_3_]_2_[Mo(CN)_6_].5H_2_O (Jun & Zhang, 2010). Also, in the [O_2_P(OCH_3_)_2_]^-^ anions, the P atom is four-coordinated in a distorted tetrahedral geometry. The P—O bond lengths and angles are within normal range as observed in [Ni(H_2_O)_6_](DMP)_2_ (Rafizadeh & Amani, 2006*a*). In the crystal, intermolecular N—H···O and C—H···O hydrogen bonds form a three-dimensional network (Table 1, Fig. 2).

### Experimental

For the preparation of the title compound, en (0.40 ml, 6.0 mmol) was added to a solution of [Ni(H_2_O)_6_](DMP)_2_ (0.83 g, 2.0 mmol) in DMSO (10 ml) and the resulting violet solution was stirred for 2 h at room temperature. This solution was left to evaporate slowly at room temperature. After 2 months, violet block crystals of the title compound were isolated (yield: 0.71 g, 72.6%).

### Refinement

H atoms on C atoms were positioned geometrically and refined as riding atoms, with C—H = 0.97 (CH_2_) and 0.96 (CH_3_) Å and with *U*_iso_(H) = 1.2*U*_eq_(C). H atoms on N atoms were located from a difference Fourier map and refined isotropically, except those on N6. They were refined as riding atoms, with N—H = 0.90 Å and *U*_iso_(H) = 1.2*U*_eq_(N).

### Crystal data

**Table:**

[Ni(C_2_H_8_N_2_)_3_](C_2_H_6_O_4_P)_2_	*F*(000) = 1040
*M**_r_* = 489.08	*D*_x_ = 1.545 Mg m^−^^3^
Monoclinic, *P*2_1_/*n*	Mo *K*α radiation, λ = 0.71073 Å
Hall symbol: -P 2yn	Cell parameters from 9338 reflections
*a* = 9.2553 (5) Å	θ = 2.7–29.2°
*b* = 12.4913 (5) Å	µ = 1.12 mm^−^^1^
*c* = 18.190 (1) Å	*T* = 120 K
β = 90.156 (4)°	Block, violet
*V* = 2102.95 (18) Å^3^	0.49 × 0.40 × 0.38 mm
*Z* = 4	

### Data collection

**Table:**

Stoe IPDS-2T diffractometer	5205 independent reflections
Radiation source: fine-focus sealed tube	4555 reflections with *I* > 2σ(*I*)
Graphite monochromator	*R*_int_ = 0.031
Detector resolution: 0.15 pixels mm^-1^	θ_max_ = 29.2°, θ_min_ = 2.7°
ω scans	*h* = −12→10
Absorption correction: numerical (*X-SHAPE* and *X-RED*; Stoe & Cie, 2002)	*k* = −17→14
*T*_min_ = 0.590, *T*_max_ = 0.650	*l* = −21→24
9338 measured reflections	

### Refinement

**Table:**

Refinement on *F*^2^	Primary atom site location: structure-invariant direct methods
Least-squares matrix: full	Secondary atom site location: difference Fourier map
*R*[*F*^2^ > 2σ(*F*^2^)] = 0.034	Hydrogen site location: inferred from neighbouring sites
*wR*(*F*^2^) = 0.085	H atoms treated by a mixture of independent and constrained refinement
*S* = 1.08	*w* = 1/[σ^2^(*F*_o_^2^) + (0.0434*P*)^2^ + 1.1522*P*] where *P* = (*F*_o_^2^ + 2*F*_c_^2^)/3
5205 reflections	(Δ/σ)_max_ = 0.014
284 parameters	Δρ_max_ = 0.51 e Å^−^^3^
0 restraints	Δρ_min_ = −0.40 e Å^−^^3^

### Special details

**Table:**

Geometry. All e.s.d.'s (except the e.s.d. in the dihedral angle between two l.s. planes) are estimated using the full covariance matrix. The cell e.s.d.'s are taken into account individually in the estimation of e.s.d.'s in distances, angles and torsion angles; correlations between e.s.d.'s in cell parameters are only used when they are defined by crystal symmetry. An approximate (isotropic) treatment of cell e.s.d.'s is used for estimating e.s.d.'s involving l.s. planes.
Refinement. Refinement of *F*^2^ against ALL reflections. The weighted *R*-factor *wR* and goodness of fit *S* are based on *F*^2^, conventional *R*-factors *R* are based on *F*, with *F* set to zero for negative *F*^2^. The threshold expression of *F*^2^ > σ(*F*^2^) is used only for calculating *R*-factors(gt) *etc*. and is not relevant to the choice of reflections for refinement. *R*-factors based on *F*^2^ are statistically about twice as large as those based on *F*, and *R*- factors based on ALL data will be even larger.

### Fractional atomic coordinates and isotropic or equivalent isotropic displacement parameters (Å^2^)

**Table:**

	*x*	*y*	*z*	*U*_iso_*/*U*_eq_	
C1	0.7792 (2)	0.35678 (14)	0.35218 (10)	0.0138 (3)	
H1A	0.8421	0.2961	0.3434	0.017*	
H1B	0.6870	0.3428	0.3286	0.017*	
C2	0.7579 (2)	0.37245 (14)	0.43420 (10)	0.0142 (4)	
H2A	0.7108	0.3102	0.4552	0.017*	
H2B	0.8506	0.3818	0.4583	0.017*	
C3	0.4548 (2)	0.69465 (15)	0.39362 (11)	0.0158 (4)	
H3A	0.4030	0.7614	0.4005	0.019*	
H3B	0.4050	0.6391	0.4209	0.019*	
C4	0.4576 (2)	0.66600 (16)	0.31238 (10)	0.0162 (4)	
H4A	0.3602	0.6530	0.2948	0.019*	
H4B	0.4984	0.7246	0.2843	0.019*	
C5	0.9829 (2)	0.72847 (15)	0.40027 (11)	0.0178 (4)	
H5A	1.0827	0.7361	0.4159	0.021*	
H5B	0.9296	0.7907	0.4169	0.021*	
C6	0.9750 (2)	0.72044 (16)	0.31718 (11)	0.0186 (4)	
H6A	1.0093	0.7863	0.2951	0.022*	
H6B	1.0355	0.6621	0.3001	0.022*	
C7	0.6815 (3)	0.55279 (17)	−0.01227 (11)	0.0242 (4)	
H7A	0.7315	0.6196	−0.0064	0.029*	
H7B	0.5815	0.5621	0.0001	0.029*	
H7C	0.6891	0.5295	−0.0624	0.029*	
C8	0.9788 (3)	0.61156 (19)	0.10517 (15)	0.0301 (5)	
H8A	0.9829	0.5941	0.0538	0.036*	
H8B	1.0351	0.5608	0.1326	0.036*	
H8C	1.0169	0.6822	0.1128	0.036*	
C9	0.8392 (3)	0.79999 (16)	0.61676 (13)	0.0242 (5)	
H9A	0.7931	0.8165	0.6626	0.029*	
H9B	0.7737	0.8144	0.5770	0.029*	
H9C	0.9242	0.8434	0.6114	0.029*	
C10	0.7784 (3)	0.56998 (19)	0.76650 (11)	0.0250 (5)	
H10A	0.8726	0.6021	0.7677	0.030*	
H10B	0.7877	0.4939	0.7607	0.030*	
H10C	0.7290	0.5852	0.8116	0.030*	
N1	0.84434 (19)	0.45511 (12)	0.32143 (9)	0.0126 (3)	
H1C	0.837 (3)	0.449 (2)	0.2723 (16)	0.022 (6)*	
H1D	0.940 (4)	0.456 (3)	0.3362 (18)	0.042 (9)*	
N2	0.66754 (19)	0.46838 (12)	0.44496 (9)	0.0118 (3)	
H2C	0.682 (3)	0.491 (2)	0.4897 (16)	0.024 (7)*	
H2D	0.581 (3)	0.451 (2)	0.4390 (16)	0.026 (7)*	
N3	0.60386 (18)	0.70561 (12)	0.42125 (9)	0.0130 (3)	
H3C	0.636 (3)	0.773 (2)	0.4098 (14)	0.019 (6)*	
H3D	0.606 (3)	0.696 (2)	0.4703 (15)	0.019 (6)*	
N4	0.54660 (18)	0.56862 (13)	0.30284 (9)	0.0136 (3)	
H4C	0.565 (3)	0.557 (2)	0.2541 (17)	0.029 (7)*	
H4D	0.495 (3)	0.512 (2)	0.3150 (15)	0.025 (7)*	
N5	0.92017 (18)	0.63031 (13)	0.43245 (9)	0.0133 (3)	
H5C	0.981 (3)	0.578 (2)	0.4283 (15)	0.021 (7)*	
H5D	0.903 (3)	0.638 (2)	0.4756 (15)	0.020 (6)*	
N6	0.82331 (18)	0.70111 (12)	0.29583 (9)	0.0135 (3)	
H6C	0.8192	0.6762	0.2495	0.016*	
H6D	0.7729	0.7627	0.2979	0.016*	
O1	0.59564 (17)	0.52152 (13)	0.14823 (8)	0.0218 (3)	
O2	0.82725 (16)	0.40819 (10)	0.15670 (7)	0.0163 (3)	
O3	0.74502 (17)	0.47356 (11)	0.03552 (7)	0.0173 (3)	
O4	0.83165 (17)	0.60793 (11)	0.12947 (8)	0.0185 (3)	
O5	0.83257 (17)	0.49601 (11)	0.61469 (8)	0.0210 (3)	
O6	0.62989 (16)	0.62537 (11)	0.57626 (8)	0.0171 (3)	
O7	0.87916 (16)	0.68872 (11)	0.61572 (8)	0.0182 (3)	
O8	0.69760 (16)	0.61289 (11)	0.70610 (7)	0.0176 (3)	
P1	0.74375 (5)	0.49734 (4)	0.12224 (2)	0.01149 (10)	
P2	0.75573 (5)	0.59976 (4)	0.62379 (2)	0.01087 (10)	
Ni1	0.73359 (2)	0.587559 (17)	0.369019 (12)	0.00877 (7)	

### Atomic displacement parameters (Å^2^)

**Table:**

	*U*^11^	*U*^22^	*U*^33^	*U*^12^	*U*^13^	*U*^23^
C1	0.0156 (9)	0.0121 (8)	0.0136 (8)	0.0000 (6)	−0.0006 (7)	−0.0019 (6)
C2	0.0162 (9)	0.0140 (8)	0.0124 (8)	0.0002 (7)	−0.0028 (7)	0.0014 (6)
C3	0.0129 (9)	0.0195 (9)	0.0149 (8)	0.0036 (7)	0.0019 (8)	0.0002 (7)
C4	0.0121 (9)	0.0218 (9)	0.0147 (8)	0.0028 (7)	−0.0018 (8)	0.0016 (7)
C5	0.0148 (9)	0.0152 (8)	0.0235 (10)	−0.0036 (7)	−0.0054 (8)	−0.0002 (7)
C6	0.0146 (9)	0.0205 (9)	0.0209 (9)	−0.0031 (7)	0.0030 (8)	0.0054 (7)
C7	0.0346 (13)	0.0236 (10)	0.0144 (9)	−0.0012 (9)	−0.0034 (9)	0.0051 (7)
C8	0.0210 (11)	0.0258 (11)	0.0435 (14)	−0.0081 (9)	−0.0027 (11)	0.0073 (10)
C9	0.0250 (11)	0.0149 (9)	0.0328 (11)	−0.0060 (8)	−0.0085 (10)	0.0026 (8)
C10	0.0312 (13)	0.0319 (11)	0.0118 (9)	0.0004 (9)	−0.0040 (9)	0.0039 (8)
N1	0.0143 (8)	0.0147 (7)	0.0088 (7)	−0.0001 (6)	−0.0005 (7)	−0.0010 (5)
N2	0.0132 (8)	0.0122 (7)	0.0101 (7)	−0.0021 (6)	0.0008 (7)	−0.0006 (5)
N3	0.0140 (8)	0.0135 (7)	0.0115 (7)	0.0008 (6)	0.0008 (6)	0.0006 (5)
N4	0.0118 (7)	0.0172 (7)	0.0120 (7)	−0.0008 (6)	0.0009 (6)	−0.0006 (6)
N5	0.0122 (8)	0.0167 (7)	0.0109 (7)	0.0002 (6)	−0.0007 (6)	−0.0017 (6)
N6	0.0149 (8)	0.0135 (7)	0.0121 (7)	−0.0005 (5)	0.0008 (7)	0.0012 (5)
O1	0.0172 (7)	0.0314 (8)	0.0168 (7)	0.0059 (6)	0.0028 (6)	0.0006 (6)
O2	0.0215 (7)	0.0128 (6)	0.0145 (6)	0.0022 (5)	−0.0014 (6)	0.0010 (5)
O3	0.0269 (8)	0.0160 (6)	0.0089 (6)	0.0010 (5)	−0.0006 (6)	−0.0001 (5)
O4	0.0225 (8)	0.0123 (6)	0.0206 (7)	−0.0010 (5)	−0.0036 (6)	−0.0013 (5)
O5	0.0211 (7)	0.0174 (7)	0.0244 (7)	0.0039 (6)	0.0023 (7)	−0.0039 (5)
O6	0.0147 (7)	0.0219 (7)	0.0147 (6)	−0.0020 (5)	−0.0037 (6)	0.0006 (5)
O7	0.0129 (7)	0.0181 (6)	0.0236 (7)	−0.0030 (5)	−0.0005 (6)	0.0022 (5)
O8	0.0187 (7)	0.0240 (7)	0.0101 (6)	0.0038 (5)	0.0024 (6)	0.0011 (5)
P1	0.0146 (2)	0.0111 (2)	0.00880 (19)	0.00133 (16)	−0.00036 (18)	−0.00062 (15)
P2	0.0095 (2)	0.0136 (2)	0.0095 (2)	−0.00065 (15)	0.00064 (18)	−0.00094 (15)
Ni1	0.00858 (12)	0.00985 (11)	0.00788 (11)	−0.00036 (8)	0.00025 (9)	0.00034 (7)

### Geometric parameters (Å, º)

**Table:**

C1—N1	1.479 (2)	C9—H9C	0.9600
C1—C2	1.518 (2)	C10—O8	1.431 (3)
C1—H1A	0.9700	C10—H10A	0.9600
C1—H1B	0.9700	C10—H10B	0.9600
C2—N2	1.475 (2)	C10—H10C	0.9600
C2—H2A	0.9700	N1—Ni1	2.1313 (15)
C2—H2B	0.9700	N1—H1C	0.90 (3)
C3—N3	1.474 (3)	N1—H1D	0.93 (4)
C3—C4	1.521 (3)	N2—Ni1	2.1220 (15)
C3—H3A	0.9700	N2—H2C	0.87 (3)
C3—H3B	0.9700	N2—H2D	0.83 (3)
C4—N4	1.479 (2)	N3—Ni1	2.1272 (15)
C4—H4A	0.9700	N3—H3C	0.92 (3)
C4—H4B	0.9700	N3—H3D	0.90 (3)
C5—N5	1.478 (2)	N4—Ni1	2.1187 (18)
C5—C6	1.516 (3)	N4—H4C	0.91 (3)
C5—H5A	0.9700	N4—H4D	0.89 (3)
C5—H5B	0.9700	N5—Ni1	2.1418 (18)
C6—N6	1.476 (3)	N5—H5C	0.86 (3)
C6—H6A	0.9700	N5—H5D	0.81 (3)
C6—H6B	0.9700	N6—Ni1	2.1166 (15)
C7—O3	1.442 (2)	N6—H6C	0.9000
C7—H7A	0.9600	N6—H6D	0.9000
C7—H7B	0.9600	O1—P1	1.4824 (15)
C7—H7C	0.9600	O2—P1	1.4925 (14)
C8—O4	1.434 (3)	O3—P1	1.6052 (14)
C8—H8A	0.9600	O4—P1	1.6084 (14)
C8—H8B	0.9600	O5—P2	1.4878 (14)
C8—H8C	0.9600	O6—P2	1.4835 (16)
C9—O7	1.438 (2)	O7—P2	1.6008 (14)
C9—H9A	0.9600	O8—P2	1.6008 (13)
C9—H9B	0.9600		
			
N1—C1—C2	108.59 (14)	Ni1—N1—H1D	109 (2)
N1—C1—H1A	110.0	H1C—N1—H1D	111 (3)
C2—C1—H1A	110.0	C2—N2—Ni1	108.62 (11)
N1—C1—H1B	110.0	C2—N2—H2C	107.3 (19)
C2—C1—H1B	110.0	Ni1—N2—H2C	109.7 (18)
H1A—C1—H1B	108.4	C2—N2—H2D	108.2 (19)
N2—C2—C1	108.08 (15)	Ni1—N2—H2D	112 (2)
N2—C2—H2A	110.1	H2C—N2—H2D	111 (3)
C1—C2—H2A	110.1	C3—N3—Ni1	108.20 (11)
N2—C2—H2B	110.1	C3—N3—H3C	108.0 (17)
C1—C2—H2B	110.1	Ni1—N3—H3C	110.7 (16)
H2A—C2—H2B	108.4	C3—N3—H3D	109.8 (17)
N3—C3—C4	109.55 (15)	Ni1—N3—H3D	109.7 (16)
N3—C3—H3A	109.8	H3C—N3—H3D	110 (2)
C4—C3—H3A	109.8	C4—N4—Ni1	107.20 (12)
N3—C3—H3B	109.8	C4—N4—H4C	110.2 (19)
C4—C3—H3B	109.8	Ni1—N4—H4C	115 (2)
H3A—C3—H3B	108.2	C4—N4—H4D	109.3 (17)
N4—C4—C3	108.57 (16)	Ni1—N4—H4D	112.8 (19)
N4—C4—H4A	110.0	H4C—N4—H4D	103 (2)
C3—C4—H4A	110.0	C5—N5—Ni1	108.09 (12)
N4—C4—H4B	110.0	C5—N5—H5C	109.6 (18)
C3—C4—H4B	110.0	Ni1—N5—H5C	106.7 (19)
H4A—C4—H4B	108.4	C5—N5—H5D	111.3 (19)
N5—C5—C6	108.79 (16)	Ni1—N5—H5D	113 (2)
N5—C5—H5A	109.9	H5C—N5—H5D	108 (3)
C6—C5—H5A	109.9	C6—N6—Ni1	108.58 (12)
N5—C5—H5B	109.9	C6—N6—H6C	110.0
C6—C5—H5B	109.9	Ni1—N6—H6C	110.0
H5A—C5—H5B	108.3	C6—N6—H6D	110.0
N6—C6—C5	108.44 (15)	Ni1—N6—H6D	110.0
N6—C6—H6A	110.0	H6C—N6—H6D	108.4
C5—C6—H6A	110.0	C7—O3—P1	117.49 (12)
N6—C6—H6B	110.0	C8—O4—P1	118.86 (13)
C5—C6—H6B	110.0	C9—O7—P2	119.09 (13)
H6A—C6—H6B	108.4	C10—O8—P2	120.22 (13)
O3—C7—H7A	109.5	O1—P1—O2	119.76 (8)
O3—C7—H7B	109.5	O1—P1—O3	111.12 (9)
H7A—C7—H7B	109.5	O2—P1—O3	105.64 (8)
O3—C7—H7C	109.5	O1—P1—O4	105.48 (9)
H7A—C7—H7C	109.5	O2—P1—O4	110.20 (8)
H7B—C7—H7C	109.5	O3—P1—O4	103.54 (8)
O4—C8—H8A	109.5	O6—P2—O5	119.85 (9)
O4—C8—H8B	109.5	O6—P2—O7	110.87 (8)
H8A—C8—H8B	109.5	O5—P2—O7	104.66 (8)
O4—C8—H8C	109.5	O6—P2—O8	104.94 (8)
H8A—C8—H8C	109.5	O5—P2—O8	110.81 (8)
H8B—C8—H8C	109.5	O7—P2—O8	104.86 (8)
O7—C9—H9A	109.5	N6—Ni1—N4	92.22 (6)
O7—C9—H9B	109.5	N6—Ni1—N2	173.64 (7)
H9A—C9—H9B	109.5	N4—Ni1—N2	93.17 (7)
O7—C9—H9C	109.5	N6—Ni1—N3	92.25 (6)
H9A—C9—H9C	109.5	N4—Ni1—N3	82.53 (6)
H9B—C9—H9C	109.5	N2—Ni1—N3	91.81 (6)
O8—C10—H10A	109.5	N6—Ni1—N1	94.29 (6)
O8—C10—H10B	109.5	N4—Ni1—N1	94.33 (6)
H10A—C10—H10B	109.5	N2—Ni1—N1	81.93 (6)
O8—C10—H10C	109.5	N3—Ni1—N1	172.86 (6)
H10A—C10—H10C	109.5	N6—Ni1—N5	81.64 (6)
H10B—C10—H10C	109.5	N4—Ni1—N5	171.90 (6)
C1—N1—Ni1	107.09 (11)	N2—Ni1—N5	93.30 (7)
C1—N1—H1C	105.9 (17)	N3—Ni1—N5	92.40 (7)
Ni1—N1—H1C	116.1 (17)	N1—Ni1—N5	91.40 (7)
C1—N1—H1D	107 (2)		
			
N1—C1—C2—N2	56.9 (2)	C6—N6—Ni1—N1	−74.78 (13)
N3—C3—C4—N4	55.1 (2)	C6—N6—Ni1—N5	16.01 (12)
N5—C5—C6—N6	55.6 (2)	C4—N4—Ni1—N6	−73.62 (11)
C2—C1—N1—Ni1	−43.53 (17)	C4—N4—Ni1—N2	109.78 (11)
C1—C2—N2—Ni1	−40.33 (18)	C4—N4—Ni1—N3	18.36 (11)
C4—C3—N3—Ni1	−37.22 (17)	C4—N4—Ni1—N1	−168.09 (11)
C3—C4—N4—Ni1	−43.55 (17)	C2—N2—Ni1—N4	107.14 (13)
C6—C5—N5—Ni1	−39.95 (18)	C2—N2—Ni1—N3	−170.24 (13)
C5—C6—N6—Ni1	−42.24 (17)	C2—N2—Ni1—N1	13.21 (13)
C7—O3—P1—O1	53.70 (17)	C2—N2—Ni1—N5	−77.74 (13)
C7—O3—P1—O2	−174.96 (15)	C3—N3—Ni1—N6	102.42 (12)
C7—O3—P1—O4	−59.09 (16)	C3—N3—Ni1—N4	10.48 (12)
C8—O4—P1—O1	−177.29 (16)	C3—N3—Ni1—N2	−82.48 (12)
C8—O4—P1—O2	52.12 (18)	C3—N3—Ni1—N5	−175.86 (12)
C8—O4—P1—O3	−60.47 (18)	C1—N1—Ni1—N6	−168.38 (12)
C9—O7—P2—O6	45.21 (17)	C1—N1—Ni1—N4	−75.83 (12)
C9—O7—P2—O5	175.78 (15)	C1—N1—Ni1—N2	16.76 (12)
C9—O7—P2—O8	−67.52 (17)	C1—N1—Ni1—N5	109.90 (12)
C10—O8—P2—O6	164.87 (15)	C5—N5—Ni1—N6	13.37 (12)
C10—O8—P2—O5	34.15 (18)	C5—N5—Ni1—N2	−170.51 (12)
C10—O8—P2—O7	−78.24 (16)	C5—N5—Ni1—N3	−78.56 (12)
C6—N6—Ni1—N4	−169.29 (12)	C5—N5—Ni1—N1	107.49 (12)
C6—N6—Ni1—N3	108.10 (12)		

### Hydrogen-bond geometry (Å, º)

**Table:**

*D*—H···*A*	*D*—H	H···*A*	*D*···*A*	*D*—H···*A*
N1—H1*C*···O2	0.90 (3)	2.17 (3)	3.057 (2)	171 (2)
N1—H1*D*···O5^i^	0.93 (4)	2.36 (4)	3.263 (2)	165 (3)
N2—H2*C*···O6	0.87 (3)	2.35 (3)	3.111 (2)	146 (2)
N2—H2*D*···O6^ii^	0.84 (3)	2.19 (3)	3.015 (2)	169 (2)
N3—H3*C*···O2^iii^	0.92 (3)	2.11 (3)	2.971 (2)	157 (2)
N3—H3*D*···O6	0.90 (3)	2.13 (3)	3.002 (2)	163 (2)
N4—H4*C*···O1	0.92 (3)	2.00 (3)	2.910 (2)	176 (3)
N4—H4*D*···O8^ii^	0.88 (3)	2.40 (3)	3.205 (2)	152 (2)
N5—H5*C*···O5^i^	0.87 (3)	2.11 (3)	2.911 (2)	154 (2)
N6—H6*C*···O4	0.90	2.35	3.243 (2)	175
N6—H6*D*···O2^iii^	0.90	2.20	3.064 (2)	160
C9—H9*C*···O1^iv^	0.96	2.41	3.305 (3)	155

Symmetry codes: (i) −*x*+2, −*y*+1, −*z*+1; (ii) −*x*+1, −*y*+1, −*z*+1; (iii) −*x*+3/2, *y*+1/2, −*z*+1/2; (iv) *x*+1/2, −*y*+3/2, *z*+1/2.

## References

[bb1] Amani, V., Rafizadeh, M., Yousefi, M. & Zargar, N. S. (2006). *Anal. Sci.* **22**, x303–x304.

[bb2] Farrugia, L. J. (1997). *J. Appl. Cryst.* **30**, 565.

[bb3] Farrugia, L. J. (1999). *J. Appl. Cryst.* **32**, 837–838.

[bb4] Jun, Q. & Zhang, C. (2010). *Acta Cryst.* E**66**, m24–m25. 10.1107/S1600536809051964 PMC2980088 21579925

[bb5] Rafizadeh, M. & Amani, V. (2006*a*). *Acta Cryst.* E**62**, m1776–m1777.

[bb6] Rafizadeh, M. & Amani, V. (2006*b*). *Anal. Sci.* **22**, x211–x212.

[bb7] Rafizadeh, M. & Amani, V. (2007). *Z. Anorg. Allg. Chem.* **633**, 2738–2741.

[bb8] Rafizadeh, M., Amani, V. & Aghayan, H. (2006). *Acta Cryst.* E**62**, m2450–m2452.

[bb9] Rafizadeh, M., Amani, V. & Broushaky, M. (2006). *Anal. Sci.* **22**, x213–x214.

[bb10] Rafizadeh, M., Amani, V. & Farajian, H. (2007). *Z. Anorg. Allg. Chem.* **633**, 1143–1145.

[bb11] Rafizadeh, M., Amani, V. & Mortazavi, N. S. (2009). *Bull. Korean Chem. Soc.* **30**, 489–492.

[bb12] Rafizadeh, M., Hoseinzadeh, F. & Amani, V. (2006). *Anal. Sci.* **22**, x3–x4.

[bb13] Rafizadeh, M., Tayebee, R., Amani, V. & Nasseh, M. (2005). *Bull. Korean Chem. Soc.* **26**, 594–598.

[bb14] Sheldrick, G. M. (2008). *Acta Cryst.* A**64**, 112–122. 10.1107/S0108767307043930 18156677

[bb15] Stoe & Cie (2002). *X-AREA*, *X-RED* and *X-SHAPE* Stoe & Cie, Darmstadt, Germany.

